# Assessment of the Synergetic Performance of Nanostructured CeO_2_-SnO_2_/Al_2_O_3_ Mixed Oxides on Automobile Exhaust Control

**DOI:** 10.3390/ma15238460

**Published:** 2022-11-28

**Authors:** Varuna Jayachandran, Vishnu Shankar Dhandapani, Elango Muniappan, Dongkyou Park, Byungki Kim, A. P. Arun, P. R. Ayyappan

**Affiliations:** 1 Department of Physics, PSG College of Arts & Science, Coimbatore 641014, India; 2 Department of Electromechanical Convergence Engineering, Korea University of Technology and Education, Cheonan 31253, Chungnam, Republic of Korea; 3 School of Mechatronics Engineering, Korea University of Technology and Education, Cheonan 31253, Chungnam, Republic of Korea; 4 Department of Mechanical Engineering, Kumaraguru College of Technology, Coimbatore 641049, India

**Keywords:** air pollution, CeO_2_-SnO_2_/Al_2_O_3_, oxygen vacancies, BET, XPS, Raman, diesel exhaust, AVL gas analyzer

## Abstract

In order to control diesel exhaust emission, CeO_2_-SnO_2_/Al_2_O_3_ (CTA) mixed oxides were prepared and coated on perforated stainless steel (SS) filter plates, and the catalytic activities were analyzed in this work. The CeO_2_-SnO_2_ (different compositions of Ce/Sn—2:8; 1:1; 8:2) composites and Al_2_O_3_ were prepared separately via a co-precipitation approach, and CeO_2_-SnO_2_/Al_2_O_3_ (CTA) mixed oxides were attained by mechanical mixing of 75 wt% CeO_2_-SnO_2_ composites with 25 wt% Al_2_O_3_. X-ray diffraction (XRD) and Raman spectroscopy were performed for all three CeO_2_-SnO_2_/Al_2_O_3_ (CTA) mixed oxides; the CeO_2_-SnO_2_/Al_2_O_3_ (Ce/Sn-1:1) sample confirmed the presence of cubic and tetragonal mixed faces, which enhances the redox nature (catalytic activities). Various characterizations such as high-resolution transmission electron microscopy (HRTEM), Brunauer–Emmett–Teller (BET) analysis, X-ray photoelectron spectroscopy (XPS), and a scanning electron microscope (SEM) were employed on CeO_2_-SnO_2_/Al_2_O_3_ (Ce/Sn-1:1) sample to investigate the structural, textural, compositional, and morphological properties. The CeO_2_-SnO_2_/Al_2_O_3_ (Ce/Sn-1:1) sample was coated on a perforated stainless steel (SS) filter plate via a simple, cost-effective, and novel method, and an exhaust emission test for various compression ratios (CR), injection pressure (IP), and load (L) was completed using an AVL Digas analyzer. The CeO_2_-SnO_2_/Al_2_O_3_ (Ce/Sn-1:1) sample, with a size of 10.22 nm and a high surface area of about 73 m^2^ g^−1^, exhibit appreciable catalytic properties.

## 1. Introduction

India is the most populated country, with around 1.3 billion people sprawled across various cities, towns, and rural clusters, and is going through rapid economic growth. India faces many economic and environmental concerns, and air quality is one of them. The air quality has degraded since the 1990s [1]. The Indian government introduced stringent norms to regulate the air quality standards in the name of the Bharat Stage (BS). Currently, the government enforces BS-VI by hopping BS-V, which results in a reduction in NOx concentration in petrol and diesel engines by 25% and 68%, respectively. Moreover, this regulation will cut 82% of the particulate matter (PM) content [2]. Chennai is one of the metropolitan cities in India, with stupendous industrial sectors, facing 43% of its air pollution from transport and 14% from diesel generator sets [3].

Developing sustainable fuels and catalysts to deteriorate vehicular emissions to improve the air quality are the possible solutions to minimize the alarming stage of air pollution. In 1970, catalytic converters came into existence to treat automobile-soot pollutants. Ceria–zirconia oxides are the usual metal supports coated on a ceramic monolith, which act as oxidation and reduction catalysts because of their redox behavior [4]. Using precious metals such as platinum, palladium, and rhodium increases the catalytic converter cost yet quickly sinters even with metal supports, *which deactivates the catalytic activity* [5]. Adding alumina avoids the sintering of these oxides and facilitates the availability of surface-active oxygen species to endure particle sintering and catalyst deactivation [6]. Morikawa et al. (2008) [7] validated that alumina addition on a nanometer scale hinders particle growth upon heat treatment, by the concept of “barrier diffusion”. Nanosized catalysts provide high surface area, bulk oxygen ion conduction, durability, structure, and thermal stability. In addition to their outspoken features, their textural and chemical parameters are variable. The robust metal–oxygen network renders its role in soot oxidation as quite fundamental. The diverse metal–oxygen interactions on the surface are accountable for the surface structures. In selective partial oxidation processes, the intrinsic metal–oxygen composition and defect orientation are significant. The structure and chemical selectivity of the soot redox reactions assist in understanding the catalytic abilities of the catalysts [8]. XiaolinGuO et al. (2016) prepared Cu_2_O as the catalyst for CO oxidation, which occurs rapidly over Cu^0^, accompanied by Cu^+^ and Cu^2+^ species. In an O_2_-rich environment, the Cu_2_O catalyst is more active than in the O_2_-lean condition [9]. Tin (Sn) possesses two stable oxides, namely SnO and SnO_2_. The Sn^3+^ valence state is unavailable due to the idle pair effect (5p25s2 electron configuration) in Sn. Substituting Sn in CeO_2_ gives CeO_2_-SnO_2_ enhanced catalytic activity. A Sn^4+^ to Sn^2+^ redox couple affords a two-electron exchange, which substantially elevates the oxygen storage capacity (OSC) compared to Zr^4+^ in CeO_2_-ZrO_2_ [10]. X.D. Wu et al. (2011) [11] witnessed that contact between catalysts and soot impacts catalytic performance. L. Hensgen et al. (2011) [12] proposed that the active oxygen species O* (O^2−^, O^−^, O_2_^−^) generated must travel a distance of about ~3 nm from bulk to catalyst surface and become desorbed to react with soot particles. B. Bassou et al. (2010) [13] claimed that the H_2_ reduction test is not reliable to detect the reduction property of the catalysts, since the H_2_ molecules have far greater reduction capacity than the soot particles.

Many literature reviews shed some light on the exciting properties of the solid solutions of CeO_2_-SnO_2_/Al_2_O_3_ catalysts, especially on their thermal stability against sintering and durability. This paper focuses mainly on the mixed-phase compositions obtained by many authors who have validated the solid-solution catalysts as the best for automobile-soot treatment. We have attempted to justify the microstructural, textural, and chemical properties on a nanodimensional scale to emphasize the catalytic behavior of the prepared samples. We have also used a novel technique to coat the catalysts inside the catalytic converter to design them at a relatively low cost.

## 2. Materials and Methods

### 2.1. Chemicals

Nitrate salts of cerium (Ce(NO_3_)_3_.6H_2_O, 99%), Tin (Sncl_4_.5H_2_O, 99%), and aluminum (Al(NO_3_)_3_.9H_2_O, 99%) were obtained from Sigma Aldrich. Ammonia (NH_4_OH, 25 wt%) and Ethanol (C_2_H_5_OH) was purchased from MERCK. We have used all chemicals as received without further purification in this work.

### 2.2. Synthesis Route

CeO_2_-SnO_2_/Al_2_O_3_ (CTA) samples of different compositions of Ce/Sn = 2:8; 1:1; 8:2 were prepared with constant alumina composition of 25 wt%. CeO_2_-SnO_2_ oxide was synthesized using the co-precipitation technique. Mixed solution of Ce(NO_3_)_3_ and Sncl_4_ salts was attained by adding equal volumes of ethanol and distilled water (Bisolvent). The solution was kept stirred until a clear solution was acquired. Once the precursors were completely dissolved, precipitation was commenced by injecting NH_4_OH solution into the growth medium under vigorous stirring. The pH of the growth medium was increased to 10 by proper addition of NH_4_OH at room temperature, which provides suitable conditions for the formation of M-OH (M=Ce, Sn). The precipitate was kept under continual stirring for about 7 h and aged overnight. The precipitate was centrifuged for 10 min at 3000 rpm and washed multiple times with distilled water and organic solvents such as acetone and ethanol. Then, the precipitates were calcined in a muffle furnace at 500 °C for 5 h, and, thus, CeO_2_-SnO_2_ oxides were synthesized [14].

Al_2_O_3_ nanoparticles were prepared using the method mentioned above, and 25 wt% of them were mechanically mixed with CeO_2_-SnO_2_ oxides. The samples were called CTA-28, CTA-55, and CTA-82 based on their Ce/Sn ratios of 2:8, 1:1, and 8:2, respectively.

### 2.3. Catalyst Substrate Preparation

The perforated stainless steel (SS) substrates were first cleaned in liquid soap in an ultrasonic bath for 30 min and then in acetone under the same circumstances; each step was followed by a drying phase at 100 °C for 2 h. Amidst that, the substrates were pre-treated to create a rough surface that would aid the adhesion of the catalyst to the substrate. The emery paper was used to etch the substrate surface, which was subsequently cleaned with distilled water and acetone. It was then dried for around 30 min in a hot air oven at 50 °C.

**Figure 1:**
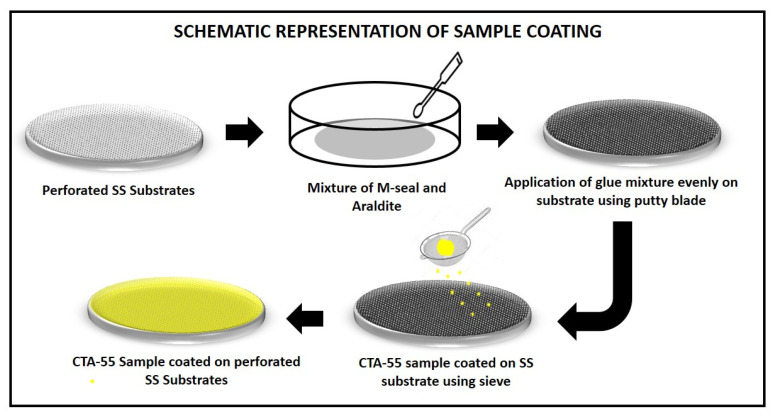
Schematic representation of CTA-55 sample-coating technique.

The substrates were coated with the prepared CTA-55 nanoparticles with the help of M-seal, and Araldite acts as reduction and oxidation catalyst, as shown in Figure 1. The perforated SS substrates were painted with an equal mixture of M-seal and Araldite. Then, the nanoparticles were evenly coated over the glued surface of the substrate using a sieve. This work aims to reduce the cost of catalytic converters, so we used cost-effective novel techniques to test the emission control.

### 2.4. Sample Characterization

“The microstructural properties and phase purity were examined using an X’Pert Pro diffractometer with a CuKα (1.5406 Å) source, BRUKER RFS 27 Standalone FT-RAMAN spectrometer and Japan-made JEOL-JEM (2010) HRTEM microscope with a BRUCKER QUANTAX 200-Z10 EDX detector. The textural analysis done by BELSORP Mini II. Surface analysis was performed by X-ray photoelectron spectrometer (XPS), a Versa Probe-III (Physical Electronics). The surface morphology was examined using a TESCAN MIRA-3 Scanning Electron Microscope (SEM) along with an EDAX detector”.

### 2.5. Catalyst Performance Testing

The Kirloskar diesel test engine with the gas analyzer was loaded with our catalytic converter to examine the catalytic activity. The engine specifications are tabulated in detail in Table 1. The four-stroke single-cylinder diesel engine was operated at an optimum temperature with an eddy current dynamometer. The direct-injection engine has a variable compression ratio of 17.5:1 to 20:1, with maximum torque of 235 Nm. In the present work, we examined the catalytic conversion for three compression ratios (CR), injection pressures (IP), and loads (L), with and without filter (control).

## 3. Results

### 3.1. Structural Investigations (XRD, Raman, HRTEM)

**Figure 2:**
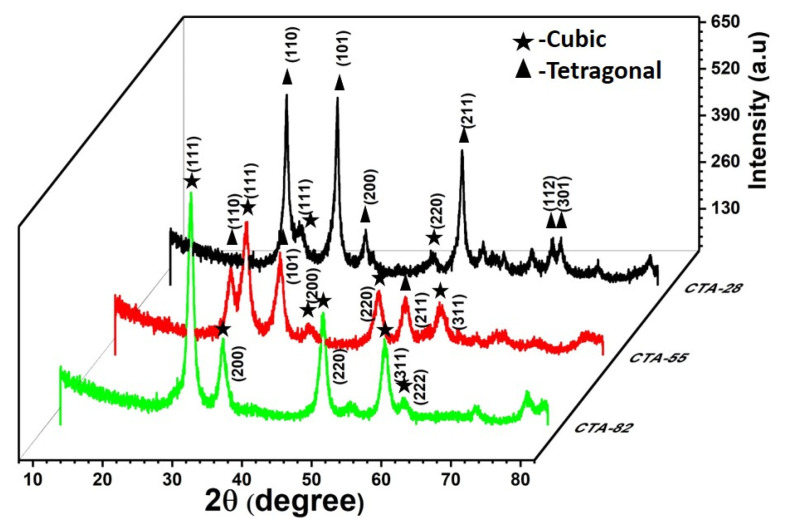
XRD patterns ofCTA-28, CTA-55, and CTA-82.

X-ray diffraction pattern of the prepared samples calcined at 500 °C were shown in Figure 2. The CTA-28 sample displays the diffraction peaks at (110), (101), (200), (211), (220), (112), and (301) of the tetragonal rutile (SnO_2_–JCPDS–41-1445) crystal structure and space group–P4_2_/mnm, whereas the (111) and (220) planes correspond to the cubic fluorite phase (CeO_2_ JCPDS–34-0394) and space group–F*m*3*m* [15,16]. The miller planes in CTA-55 appear with a slightly higher angle shift upon increasing the Ce content (2θ = 26.62, 28.57, 33.90,47.71, 51.79). The peak shift suggests considerable interaction between Ce and Sn species over the Ce–Sn oxide catalysts. Further increasing the ratio of Ce/Sn in CTA-82, the peaks attributed to CeO_2_ undergoes peak broadening, and the absence of SnO_2_ peaks was primarily caused by two reasons. They might exist in the amorphous form or the Sn^4+^ cations may have effectively diffused inside the CeO_2_ lattice to create a homogeneous solid solution (-Ce^4+^–O–Sn^4+^ species) analogous to the literature [17,18]. The absence of the distinctive Al_2_O_3_ peak implies that the Al^3+^ ions are well-distributed and too small to be identified by the XRD technique, since they are added by the mechanical mixing method, as stated by Li Lan et al. [19]. The peak intensity of the (111) plane increases by increasing the Ce/Sn ratio. Fronzi et al. infers that under “oxygen-rich” environments, and the stoichiometric (111) plane is anticipated to be the most stable among the surfaces investigated. The stoichiometric (111) face with sub-lattice oxygen vacancies becomes the most thermodynamically stable face under lean circumstances [20]. The crystallite size and lattice strain were calculated from the relation given below [21],

(1) $$D=\frac{K\lambda }{\beta \;Cos\theta }$$

(2) $$\varepsilon =\frac{\beta \;Cos\theta }{4}$$

The crystallite size of the (111) plane from the Debye–Scherrer relation was found to be 12.27, 10.22, and 11.41 nm for CTA-28, CTA-55, and CTA-82, respectively. The calculated lattice parameter and lattice strain are tabulated in Table 2. The lattice parameters of all the prepared samples were found to be less than that of the bulk CeO_2_ (5.423 Å), which might be due to the incorporation of Sn^4+^ cation (0.71 Å), with an ionic radius that is much smaller than that of the Ce^4+^ ion (0.92 Å) [21]. The lattice expands by increasing the Ce/Sn ratio, which might be caused by a more significant Ce^4+^ reduction. The lattice strain increases by increasing the Ce/Sn ratio, unveiling the Sn^4+^ interactions with Ce^4+^ that generally happen due to vacancy generation and the interstitial or substitutional positions taken up by atoms inside the unit cell [22]. The XRD pattern of CZA-55 reveals the existence of the cubic and tetragonal phases that show an intense synergetic impact on the electronic structure of the lattice and the subsurface oxygen atoms promoting the redox reactions. The presence of the cubic–tetragonal interface was confirmed from the calculated lattice parameters. The interfaces were anticipated to exist in CZA-55 oxides by interconnecting the shared lattice sites of the cubic and tetragonal phases, which exhibit some unique redox properties, thus improving the catalytic activity [23]. The microstructural properties of the CTA-55 sample are well-suited for catalytic applications.

**Figure 3:**
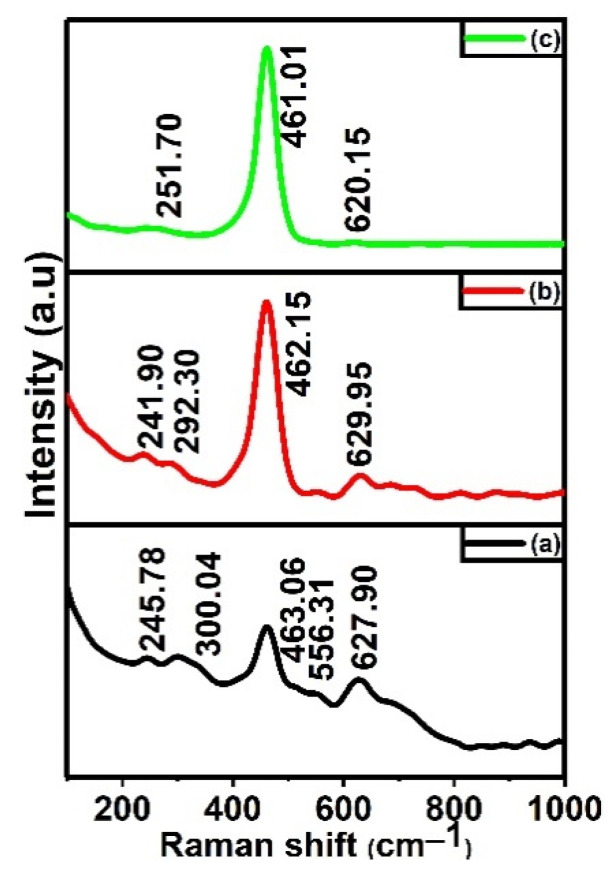
Raman spectra of Ce_x_Sn_1-x_O_2_/Al_2_O_3_ mixed oxides. (**a**) CTA-28, (**b**) CTA-55, and (**c**) CTA-82.

Raman spectra give more detailed structural information than XRD, since it is a bulk characterization. Figure 3 displays the Raman spectra of the prepared samples; the bands are below 100 cm^−1^, which depict the existence of the polymorphism or the strong lattice vibrations in the samples. The cubic fluorite phase of CeO_2_ was observed around 460–464 cm^−1^, which is the symmetric breathing mode of oxygen atoms around cerium atoms, and it has triply degenerated the F_2g_ Raman active mode [24]. Bands nearly 300 cm^−1^ represent the displacement of oxygen ions to tetragonal sites from their ideal positions, leading to partial distortion of the cubic symmetry [25]. In CTA-28 and CTA-55, the bands observed around 245, 630, and 770 cm^−1^ are attributed to the E_u_ band in the transverse optical, A_1g_, and B_2g_ modes [26]. In the CTA-82 sample, the band at 620 cm^−1^ is due to oxygen vacancy created by the inclusion of the Sn^4+^ ion in the CeO_2_ systems. By increasing the Ce/Sn ratio from 20% to 50%, the intensity of the F_2g_ band increased, implying that a simple co-precipitation technique achieves the growth of the cubic and tetragonal phases together. The incomplete phase transition from the cubic to the tetragonal phase in CTA-55 is confirmed by the presence of four active Raman bands instead of six. Due to this incomplete transition, the metal–oxygen bond is lengthened and shifts the oxygen ions to octahedral sites, which suppress the energy barrier for the subsurface oxygen ion conduction to the surface [27]. The presence of the intrinsic defective band (oxygen vacancy due to the non-stoichiometric nature of CeO_2_) and the induced defective band caused by the insertion of Sn^4+^ ensures oxygen ion mobility [28]. Thus, SnO_2_ enhances the oxygen vacancy concentration, which promotes the redox reactions. These bands are more prominent in the CTA-55 sample, and their spectra complement the XRD analysis.

**Figure 4:**
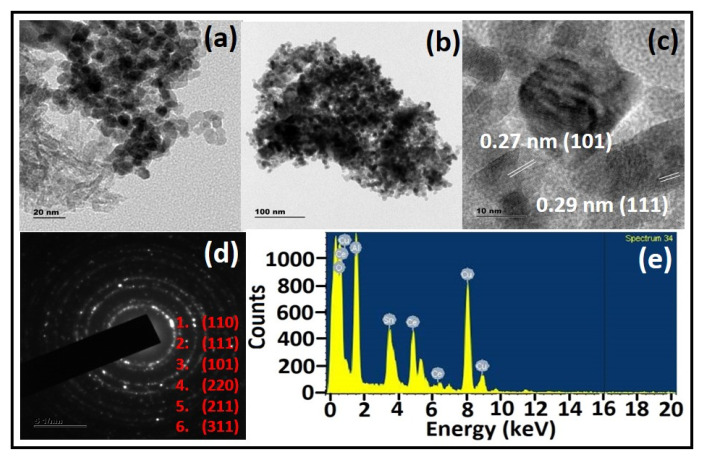
(**a**–**c**) HRTEM images of CTA-55, (**d**) SAED pattern of CTA-55, and (**e**) EDS spectra of CTA-55.

The HRTEM technique is used to discern the morphology and crystalline nature of the prepared sample. Figure 4a,b show that the sample was made of slightly agglomerated particles of irregular shapes with a mesoporous framework [29]. This agglomeration might be due to the high surface energy of the particles that undergo Oswald’s ripening process to achieve stability. This process promotes the particles with active surface sites for catalytic performance. Figure 4c witnesses the periodicity of the lattice fringes with an interplanar spacing of 0.27 and 0.29 nm observed for the (101) and (111) planes of Sn and Ce, respectively [30]. The amorphous area in this micrograph might be the presence of Al_2_O_3_ nanoparticles, which is evidenced by the dispersion of alumina inside CeO_2_-SnO_2_ systems [31]. The rings in the selected area’s electron diffraction (SAED) pattern (Figure 4d) are associated with the (110), (111), (101), (220), and (211) cubic and tetragonal rutile planes of CeO_2_ and SnO_2_. These rings indicate the polycrystalline nature of the samples that confirms the multifaceted growth [32]. Figure 4e shows the elemental composition spectra confirming the presence of Ce, Sn, Al, and O. The HRTEM results accord well with the XRD and Raman findings.

### 3.2. Textural Investigations

**Figure 5:**
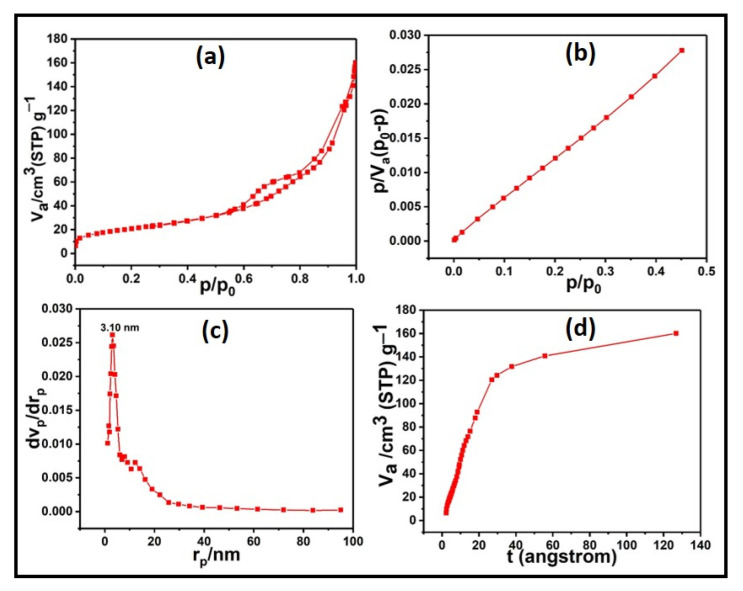
(**a**,**b**) Nitrogen-sorption isotherm of CTA-55 mixed oxide, (**c**) BJH plot, and (**d**) t-plot.

The N_2_-physisorption isothermal plots and their corresponding pore-size distribution BJH plot are shown in Figure 5. Figure 5a exhibits the isotherm of classical type IV, as specified by IUPAC, which is a typical mesoporous material. At a high relative pressure of (P/P_0_) = 1, the sample reveals a well-defined H2-type hysteresis loop with a descending adsorption branch and a reasonably steep desorption branch [33]. The wormhole-like mesostructure developed by nanoparticles is reliable with the TEM micrograph. The obtained specific surface area of the sample was found to be about 73 m^2^ g^−1^, prepared via a co-precipitation approach. On introducing SnO_2_ and Al_2_O_3_ to the CeO_2_ lattice, the surface area of the sample is increased. The large surface area increases the chance of contact between the catalyst and the soot molecules, which is beneficial for catalytic performance. Caixia Liu et al. reported that the surface area of the sample obtained via the co-precipitation route was 79.6 m^2^ g^−1^, which is comparatively higher than that of other synthesis approaches. They have achieved this surface area for a single-phase structure [34]. In the present work, the sample has attained a surface area of nearly 73 m^2^ g^−1^ for a mixed-phase composition. The mechanical mixing of the alumina particles into the CeO_2_–SnO_2_ oxides may have collapsed the pore structures, restricting us from getting a considerably larger one [35]. The BJH plot (Figure 5c) yields a single sharp, narrow peak centered at 3.4 nm, explaining that the material has a homogeneous, mesopore size distribution. The mesoporous characteristics of the sample were further confirmed through a t-plot, as shown in Figure 5d, since the positive intercept of the plot is absent. This t-plot implies the absence of micropores. The t-plot was drawn using the relation [36],

(3) $$t\;\left(angstrom\right)={\left\{\frac{13.99}{log\;\left(\frac{P_{0}}{P}\right)+0.034}\right\}}^{\frac{1}{2}}$$

As BET infers, the catalyst surfaces are mesoporous, with significant thermal stability and compositional consistency, optimizing the redox property for automobile-soot treatment on a vast scale.

### 3.3. Chemical States Analysis (XPS)

**Figure 6:**
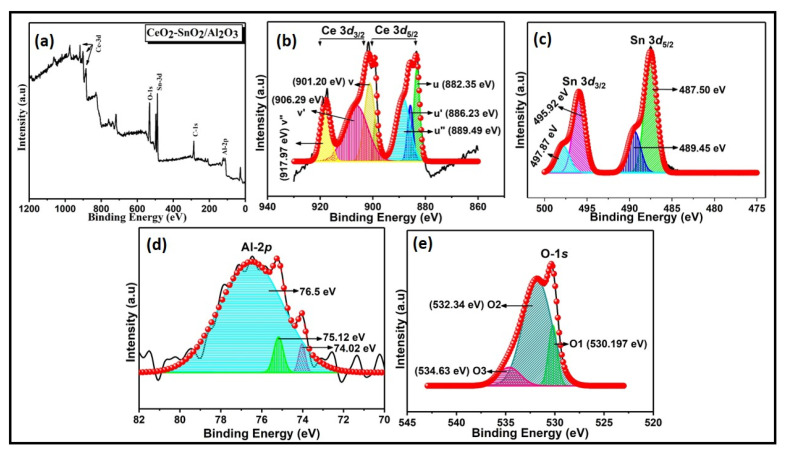
(**a**). XPS survey spectra of CTA-55 and (**b**) core-level spectra of Ce-3*d*, (**c**) Sn-3*d*, (**d**) Al-2*p*, and (**e**) O-1*s*.

XPS analysis scrutinizes the elementary oxidation state and surface compositions to learn about the nature of interactions among the metal oxides. The sample may get damaged due to charging during XPS examination, so the binding energy scale was calibrated using a standard carbon C-1s peak (285 eV). The oxidation states of Ce-3*d*, Sn-3*d*, Al-2*p*, and O-1*s* were presented in Figure 6a–e and Table 3. Figure 6a–e show the XPS survey scan and high-resolution scan of the Ce-3*d*, Sn-3*d*, Al-2*p*, and O-1*s* elements.

Figure 6b’s XPS survey scan reveals the binding energies of Ce, Sn, Al, and O, without any other peaks that indicate the high quality of the samples prepared. Six peaks were obtained by employing a Gaussian-peak-fitting deconvolution of the Ce-3*d* spectra. The core-level spectra of Ce-3*d* possess the binding energy peaks situated at 882.3 eV and 901.2 eV, corresponding to the Ce 3*d*5/2 and Ce-*3d3*/2 states of Ce^4+^ cation, respectively. These peaks were labeled as ‘u’ and ‘v’, respectively, presenting in the electronic hybridization state Ce (IV) 3*d*^9^ 4*f^2^*O 2*p*^4^ [30]. The binding energy peak is centered at 886.2 eV and 906.2 eV, corresponding to the Ce-3*d*5/2 and Ce-3*d*3/2 states of Ce^3+^ cation, respectively. These peaks were labeled as u’ and v’,; respectively, presenting the electronic hybridization state Ce (III) 3*d*^9^ 4*f^2^* O 2p^5^ [30]. This splitting is due to spin-orbit coupling. The spin-orbit doublets u” (889.49 eV) and v” (917.97 eV) are assigned to the Ce (IV) 3*d*^9^ 4*f*^1^ O 2*p*^5^. These “shake-down” satellites were generated because of the charge transfer between Ce (4*f*) and O (2*p*) through primary photoemission [37]. The overlapped peaks in the spectra denote the coexistence of the Ce^4+^ and Ce^3+^ states of CeO_2_. The Ce^3+^ concentration was obtained from the relation [38].

(4) $$Ce^{3+}\left(\%\right)=\frac{Ce^{3+}}{Area\;of\;\left(Ce^{3+}+Ce^{4+}\right)}\times 100\%$$

The Ce^3+^ ratio confirms the reducibility of ceria. Figure 6c represents the Sn-3*d* core-level spectra. The binding energy of Sn-3d located at 487.5 eV and 495.9 eV corresponds to Sn-3*d*5/2 and Sn-3*d*3/2, respectively. On deconvolution, the peaks at 489.4 eV and 497.8 eV were obtained. These peaks attribute to Sn^4+^ and Sn^2+^ states inside the catalyst. The redox equilibrium depicts the synergistic interaction between Ce and Sn:

(5) $$2Ce^{3+}+Sn^{4+}↔2Ce^{4+}+Sn^{2+}$$

The spectra confirm the absence of the Sn^0^ state [39]. Figure 6e displays the O-1*s* core-level spectra. The oxygen O-1*s* band dissociates into lattice oxygen (Oβ) and adsorbed oxygen (Oα). CeO_2_-SnO_2_ catalysts have a substantially higher Oα/(Oα + Oβ) value than those of CeO_2_ and SnO_2_ [40]. The increased mobility of immense Oα concentration may strengthen oxidation reactions and improve catalytic performance due to the coexistence of CeO_2_ and SnO_2_ [41]. This implies that Sn^4+^ insertion initiates the transition of Ce^4+^ to Ce^3+^, which results in higher oxygen vacancy species and unsaturated chemical bonds on the catalyst surface in the chemisorbed oxygen on the surface [42]. Other catalysts have a lower concentration of Oα species on the surface than the CeO_2_-SnO_2_ catalysts produced via co-precipitation. The Al-2*p* core-level spectra (Figure 6d) possess a peak of 76 eV (Al^3+^), which agreed well with the NIST database [43]. These results affirm that the presence of the redox couples, Ce^4+^/Ce^3+^ and Sn^4+^/Sn^2+^, facilitates the automobile-soot treatment.

### 3.4. Morphological Analysis

**Figure 7:**
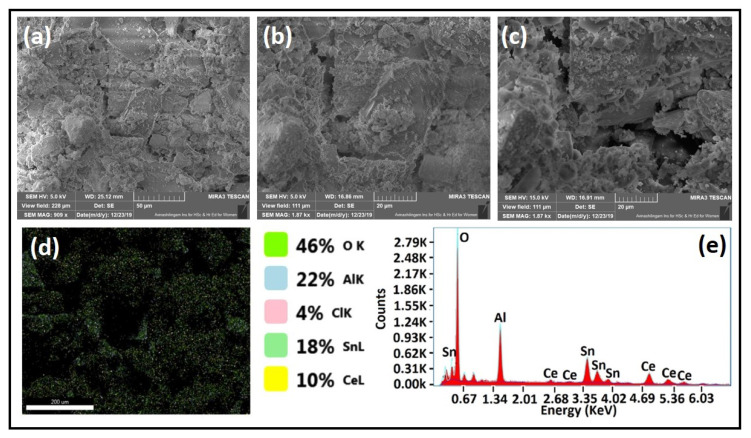
(**a**–**c**) SEM micrographs of CTA-55, (**d**) elemental mapping of CTA-55, and (**e**) EDS spectra of CTA-55.

SEM was used to study the surface morphology of the CTA-55 catalytic support. As seen in Figure 7a–c, the particles are agglomerated and asymmetric. The additional surface energy obtained during nucleation may cause the particles to accumulate. Particle orientation and stability were linked to Oswald’s ripening process [44]. Furthermore, as confirmed by BET analysis, the micrographs show that the particles were porous. The elemental mapping spectra (Figure 7d) portray that the CeO_2_, SnO_2_, and Al_2_O_3_ nanoparticles were uniformly distributed. The predicted elemental signals of Ce, Sn, Al, and O were ascribed to the chemical composition determined from the EDS spectra, as shown in Figure 7e. The Carbon signal was accomplished from the carbon tape that was used to hold the sample for analysis.

### 3.5. Catalyst Activity Test

Among the samples prepared, the structural investigations of the CTA-55 sample unveils the cubic–tetragonal phase composition with a smaller particle size of 10.22 nm and a large surface area and pore volume of 73 m^2^ g^−1^ and 0.2168 cm^3^ g^−1^ respectively. The lattice sharing of the two phases possesses unique chemical properties by improving the lattice and/or sublattice oxygen ion migration toward the surface, to readily participate in the reduction–oxidation process with the diesel exhaust pollutants.

Thus, the CTA-55 sample was coated on perforated stainless plates in the exhaust manifold to study the catalytic performance on limiting the diesel exhaust. The efficiency of the prepared catalysts was evaluated by examining the conversion rates of carbon monoxide (CO), hydrocarbon (HC), carbon dioxide (CO_2_), oxygen concentration (O_2_), and oxides of nitrogen (NO_x_). The catalytic converter is attached to the exhaust manifold of the Kirloskar diesel engine. The exhaust gas temperature was monitored, and the exhaust gas from the catalytic converter outlet was analyzed using an AVL DiGas Analyzer. This type of measurement is the universal standard test method for the pollution test [45]. In the conventional catalytic converter, the exhaust manifold creates the backpressure, from emission during the highest compression ratio, which causes the failure of the catalyst. In this work, the stacking of perforated substrates with a 15° angle shift of perforation in each substrate reduces the backpressure and promotes the oxidation reaction. The test was carried out by varying CR, IP, and load. The first test cycle was accomplished by tuning the compression ratios to 17.5, 18.75, and 20 with a constant IP at 190 bar and a load of 10 Nm. The readings were noted without a filter (taken as the control) and with a filter for comparison.

**Figure 8:**
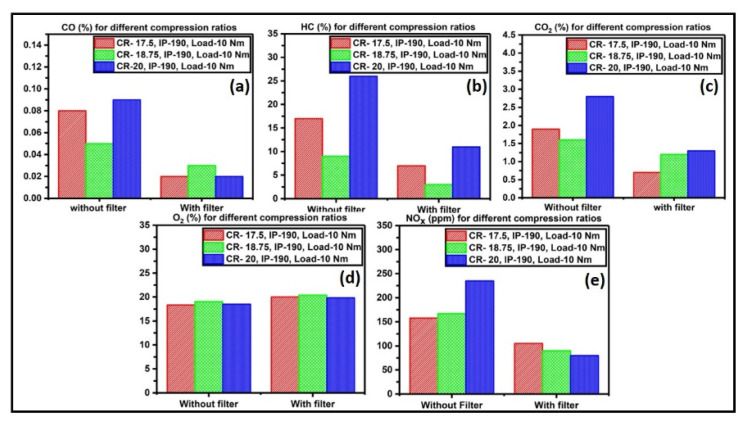
(**a**) CO emission, (**b**) HC emission, (**c**) CO_2_ emission, (**d**) NO_x_ emission, and (**e**) O_2_ emission recorded when CR = 17, 18.75, and 20; IP = 190 bar; load = 10 Nm.

Figure 8a–e display the exhaust-emission report for the control and catalyst as a filter. Figure 8a gives the CO emission results: the filter reduces the CO emissions. When the compression ratio was raised from 17.5 to 20, the CO emission elevated, but the catalyst restricted the CO emission to 75% less than that of the control emission. From Figure 8b, the HC emission for control was reduced by 60% compared to the CTA-55 sample. The HC was oxidized to CO_2_ and H_2_O vapors. Figure 8c shows the CO_2_ emissions of the engine were reduced 63%. In Figure 8d, the NOx emissions are displayed. The O_2_ concentration increases to 10% due to the catalyst’s supporting contribution in the redox cycles, as seen in Figure 8e.

**Figure 9:**
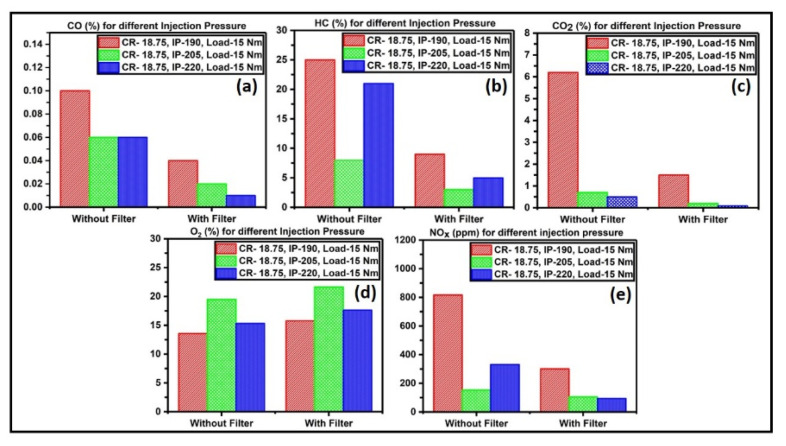
(**a**) CO emission, (**b**) HC emission, (**c**) CO_2_ emission, (**d**) NO_x_ emission, and (**e**) O_2_ emission recorded when CR = 18.75; IP = 190, 205, and 220 bar; load = 10 Nm.

Figure 9a–e exhibit the emission results of the second test cycle accomplished by varying the injection pressures to 190, 205, and 220 bar, while keeping the CR and load at constant values of 18.75 and 15 Nm, respectively. The lower IP value of 190 bar shows the higher emissions, but the catalyst filters drastically reduce the emissions. The emission reduction in CO, HC, CO_2_, and NO_x_ in this test cycle was 82%, 62%, 83%, and 47%, respectively. These reductions were good only at higher IP values. So when the IP is high, it facilitates the redox mechanisms of the exhaust soot.

**Figure 10:**
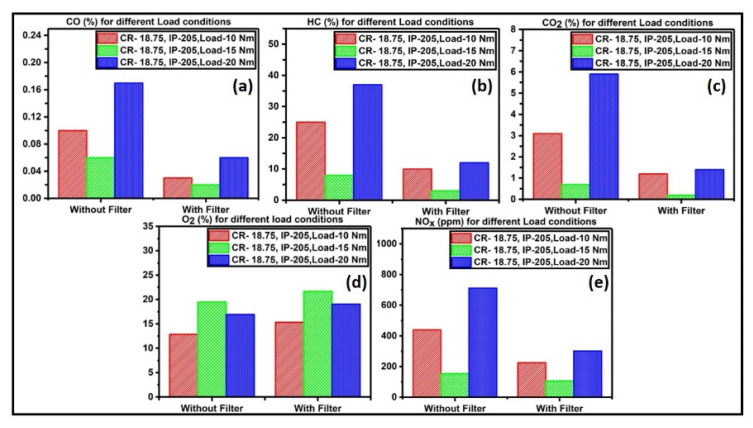
(**a**) CO emission, (**b**) HC emission, (**c**) CO_2_ emission, (**d**) NO_x_ emission, and (**e**) O_2_ emission recorded when CR = 18.75; IP = 205 bar; load = 10, 15, and 20 Nm.

Figure 10a–e present the emissions of the third test cycle taken by varying the load to 10, 15, and 20 Nm, whereas the CR and IP are at 18.75 and 205 bar, respectively. For part of the load operations, the emissions were not too high, but the emissions were on a massive scale for the full-load conditions.

Figure 8e, Figure 9e, and Figure 10e define the O_2_ emission graph. The O_2_ content in these graphs was high, which indicates the successful conversion of NO_x_ into N_2_ and O_2_. Moreover, the emissions were treated by the availability of the surface-active oxygen species, which readily react with the soot molecules. The oxygen vacancy generation is due to the charge imbalance created by the Ce^4+^ reduction. This reduction promotes the migration of oxygen ions from the bulk to the surface and interacts with the soot [46]. SnO_2_ interacts with CeO_2_ and forms the redox couples Sn^4+^/Sn^2+^ that enhance the soot treatment. The presence of Al_2_O_3_ protects the sample from sintering to avoid the catalyst deactivation of the sample [47]. S. Dey et al. [48] used Cu_2_O as a catalyst, suggesting that Cu-O-Cu species react with CO in the mixed environment, activating the lattice oxygen nearby through electron transfer. The exposed Cu and oxygen vacancy might serve as CO adsorption and O_2_ activation sites. The CO adsorbs the Cu (Cu-O-CO) and effortlessly combines with the active oxygen species to generate CO_2_. The surface oxygen that has been consumed can be replenished via mass migration in the catalyst microstructure and gaseous oxygen from defective oxygen near the Cu species. In the present work, the surface of the sample is assumed as -Ce^4+^-O-Ce^4+^-O-Sn^4+^-O-Sn^4+^; the CO molecules become adsorbed to Ce^3+^ sites, creating the oxygen vacancies. These CO molecules combine with the reactive oxygen species to form CO_2_. Similarly, the attached NO species become reduced to N_2_ by forming intermediates of many NO combinations. With the slight increase in the exhaust temperature in the manifold, the conversion efficiency is also increased [49,50]. The catalyst generates three different oxygen vacancies due to Ce^3+^, Sn^2+^, and Ce^3+^-O_v_-Sn^2+^. The Sn^2+^ cannot adsorb the CO molecules because of the steric effect created by Ce^3+^ during the NO dissociation on oxygen vacancy. The surface oxygen vacancies of Ce^3+^ and Sn^2+^ involves the soot treatment, but the oxygen vacancy of Ce^3+^-O_v_-Sn^2+^ actively contributes to the soot treatment [51]. The emissions were considerably declined by the catalyst support systems, ultimately hindering air pollution.

## 4. Conclusions

The catalytic performance of CeO_2_-SnO_2_/Al_2_O_3_ mixed oxides that coated the perforated SS filter plates were analyzed and discussed in detail. A simple and facile co-precipitation route was employed to prepare the CeO_2_-SnO_2_/Al_2_O_3_ samples at different proportions, and a cost-effective novel technique was adopted to coat them on SS filter plates. The mixed cubic and tetragonal phases in the CTA-55 sample were confirmed through the structural investigations accomplished via XRD, Raman, and HRTEM. This mixed-phase composition elucidates better reactivity towards the reactant soot molecules. The observed BET surface area, pore radius, and pore volume provided a better performance towards the exhaust treatment. The CTA-55 sample that coated the perforated SS filter plates employed in the exhaust emission test showed the promotion of oxygen ion diffusion from the bulk to the surface, facilitating the redox mechanism. The inclusion of Sn^4+^ inside the ceria benefits the oxygen vacancy generation more than bulk CeO_2_. The elemental composition witnesses the presence of redox couples Ce^4+^/Ce^3+^ and Sn^4+^/Sn^2+^ and the oxygen vacancy concentration generated by Ce^3+^, Sn^2+^, and Ce^3+^-O_v_-Sn^2+^. These redox couples and mixed phases enhance the soot treatment largely by creating a sufficient amount of active oxygen species. Thus, the CTA-55 sample was a potential material to be used as a catalyst in automobile-soot abatement with the novel and inexpensive coating technique, which plays a crucial role among the industrial sectors.

## Figures and Tables

**Table 1:** Kirloskar engine specifications.

Engine Specifications	
Make/Model	Kirloskar
Engine type	Four stroke, multifuel variable compression ratio, water-cooled
No. of cylinders	Single
Combustion chamber	Direct injection (open chamber)
Type of fuel	Diesel
Bore diameter/stroke length	87.5 mm/110 mm
Speed	1500 rpm
Max torque	235 Nm
Dynamometer	Eddy current dynamometer
Compression ratio	17.5:1 to 20:1
Rated power	5 hp (5.2 kW)

**Table 2:** Microstructural properties of CTA-55.

Sample Compositions	Miller Planes	Crystallite Size (nm) ^a^	Lattice Parameter (Å) ^a^	Lattice Strain (ε) ^a^	Position of Raman Active Band F_2g_ (cm^−1^) ^b^
CTA-28	(110) (111)	13.54 12.27	a = b = 4.6825 c = 3.1699 a = b = c = 5.3626	3.10 × 10^−3^	463.06
CTA-55	(110) (111)	12.32 10.22	a = b = 4.7795 c = 3.1995 a = b = c = 5.3307	3.39 × 10^−3^	462.15
CTA-82	(111)	11.41	a = b = c = 5.4098	2.8 × 10^−3^	461.01

^a^ Determined by XRD analysis. ^b^ Determined by RAMAN spectroscopy.

**Table 3:** Surface chemistry of CTA-55.

Sample Composition	Relative Amount (%) ^a^		Ce/Sn Ratio ^a^	Ce^3+^ (%) ^a^	Defective Oxygen (%) ^a^	Surface Area, Pore Diameter, and Pore Volume ^b^
CTA-55	Ce-3d Sn-3d Al-2p O-1s C-1s	5.69 6.55 10.08 50.96 26.72	0.86	54.32	86.74	73 m^2^g^−1^ 3.10 nm 0.2168 cm^3^g^−1^

^a^ Determined by XPS spectroscopy. ^b^ Determined by BET analysis.

## Data Availability

Not applicable.
